# Narrow Linewidth Half-Open-Cavity Random Laser Assisted by a Three-Grating Ring Resonator for Strain Detection

**DOI:** 10.3390/s22207882

**Published:** 2022-10-17

**Authors:** Bing Lv, Wentao Zhang, Wenzhu Huang, Fang Li, Yongqian Li

**Affiliations:** 1 Optoelectronic System Lab, Institute of Semiconductors, Chinese Academy of Sciences, Beijing 100083, China; 2 Department of Electronic and Communication Engineering, North China Electric Power University, Baoding 071003, China; 3 Hebei Key Laboratory of Power Internet of Things Technology, North China Electric Power University, Baoding 071003, China; 4 Baoding Key Laboratory of Optical Fiber Sensing and Optical Communication Technology, North China Electric Power University, Baoding 071003, China

**Keywords:** narrow linewidth, random fiber laser, three-grating ring resonator, high-resolution dynamic strain-sensing

## Abstract

A stabilized narrow-linewidth random fiber laser for strain detection, based on a three-grating ring (TGR) resonator and half-open-cavity structure, is proposed and investigated experimentally. The half-open-cavity structure proved to provide double optical gain of erbium-doped fiber, which was beneficial to increase the photon lifetime as well as further narrow the linewidth. Meanwhile, the stability and frequency noise of narrow lasing output was improved by suppressing the competition-induced undesired residual random lasing modes with the TGR resonator. The TGR resonator is composed of a double-cavity fiber Bragg grating Fabry–Perot (FBG-FP) interferometer, a section of single-mode fiber, and a circulator. The specially designed double-cavity FBG-FP interferometer embedded in the TGR resonator acted as the strain-sensing element and improved the resolution of the dynamic strain. A stable ultra-narrow linewidth of about 205 Hz was obtained. The frequency noise was reduced to about 2 Hz/√Hz. A high dynamic strain measuring resolution of 35 femto-strain (fε)/√Hz was achieved.

## 1. Introduction

A narrow linewidth is beneficial to suppress frequency noise levels of fiber lasers and can further improve resolution in optical dynamic strain-sensing systems [1,2,3]. Random fiber lasers (RFLs) have a simple cavity configuration, low threshold, and good temporal coherence, and they are a promising alternative for narrow linewidth and low frequency noise fiber lasers [4,5,6]. Recently, RFLs have attracted considerable interest due to their unique emission characteristic and potential sensing application prospects [4,7]. In the scheme of RFL with a half-open-cavity structure, one end usually uses the backward Rayleigh scattering (RS) inside a lengthy single mode fiber (SMF) to provide random distributed feedback, and the other end usually provides point feedback. Due to the unique resonant cavity that provides double optical gain and high lasing efficiency, the RFL with a half-open-cavity structure shows obvious advantages over high-resolution point or distributed strain-sensing and are widely developed [8,9].

Compared with backward RS inside SMF, a random weak grating array (RWGA) can provide strong random feedback, so that RWGA-based RFLs have superior performances, such as a narrow linewidth, compact structure, and high optical signal-to-noise ratio (OSNR) [10,11]. Differing from the feedback mechanism of backward RS, RWGA has wavelength dependence and can strongly improve random feedback efficiency by introducing refractive index modulation, which results in a low lasing threshold combining with the high-efficiency gain provided by erbium-doped fiber (EDF) [6,12,13,14]. RWGA can be regarded as the superposition of many Fabry–Perot interferometers which introduced by weak FBGs. Usually, increasing the number as well as randomness of weak FBGs can contribute to improving the narrow linewidth of RFL [10]. However, the number of these weak FBGs etched inside RWGA is not infinite, which makes it difficult for RFLs to obtain higher randomness and narrower linewidth.

Various spectral filter techniques and components are introduced to half-open-cavity RFLs for further compressing the linewidth, improving stability, and reducing the frequency noise of RFL. For example, FBG filters [15,16], FPI filters [12], long period fiber grating [17], Phase-shifted fiber Bragg grating (π-FBG) [18] and commercial bandpass filters [19] have been demonstrated to further compress the bandwidth of the lasing spectrum for a single-wavelength operation and improve the wavelength and power stability. The above schemes show that proper optical filters matched to the wavelength of RWGA, with a flatter response, low insert loss, and narrow bandwidth, can contribute to further compress the linewidth and suppress the frequency noise of RFL.

Dynamic strain signals, such as acoustic emission, bring a lot of information with weak amplitude and a relatively broad frequency range between a few kilohertz (kHz) and tens of kilohertz, and response amplitudes ranging from hundreds of nε to sub-pε, which are usually associated to geophysical exploration and structural damage [20]. Thus, the high-resolution strain sensors are urgently demanded for weak signal detection. Compared with electromagnetic sensors, fiber-optic interferometric strain sensors [21], distributed strain sensors [22], and fiber Bragg grating strain sensors [23] have been widely developed due to their immunity to electromagnetic interference and small size. Among them, the FBG-based optical sensor is the most typical type of strain sensor due to its embeddability, high sensitivity, and flexible multiplexing capability [24]. However, the broadband spectrum of a FBG strain sensor limits the measuring resolution to several pε [25]. Narrow linewidth fiber lasers show an alternative for high-resolution strain measurement [26] and can improve resolution to sub-pε. The higher dynamic strain resolution can be expected to further reduce the frequency noise of the fiber laser strain sensor.

Narrow linewidth RWGA-based RFLs with a half-open-cavity are gradually showing a good strain-sensing performance due to their low frequency noise and low lasing threshold. In a scheme in which an RFL strain sensor is locked to a π-FBG through the frequency-shift Pound–Drever–Hall technology, a high dynamic strain resolution of 140 fε/√Hz @ 1 kHz is realized [27]. However, tens of kilometers of lengthy SMF is used to provide Brillouin gain and Rayleigh backscatter feedback, which is usually unstable as well as sensitive to ambient environmental noise and makes the actual sensing inconvenient. In a scheme of an RFL ultrasonic sensor, the strong random grating acts as a probe [28,29] and the estimated dynamic strain resolution is 720 fε/√Hz. Its response consistency is poor, because the disordered and narrow slopes in the random grating spectrum are easily affected by environmental disturbance. The high strain resolution of 280 fε/√Hz is realized in the scheme combining a random weak grating array-based RFL and a π-FBG [26,30]. However, the narrow linewidth and stability is difficult to further improve on due to the limitation of an unflattering response of the transmission peak of π-FBG, which hinders the improvement of the dynamic strain resolution.

In this paper, we demonstrated a narrow linewidth half-open-cavity RFL assisted by a TGR resonator for dynamic strain detection. The half-open-cavity structure contributed to increasing the photon lifetime and further narrower linewidth of RFL by providing the double optical gain of erbium-doped fiber. The TGR resonator was introduced to reduce the number of undesired lasing modes and suppress the competition-induced multi-spike lasing modes, which was beneficial to realize the stabilized single-wavelength lasing, further suppress the frequency noise of the RFL, and improve the resolution of the RFL dynamic strain. The especially designed double-cavity FBG-FP interferometer, embedded in the TGR resonator, acted as a filter and sensing probe. A stable ultra-narrow linewidth of about 205 Hz was obtained. The frequency noise was reduced to about 2 Hz/√Hz. A high dynamic strain measuring resolution of 35 fε/√Hz was achieved ultimately.

## 2. Experimental Setup and Principle

### 2.1. Configuration of the RFL Strain Sensor

**Figure 1:**
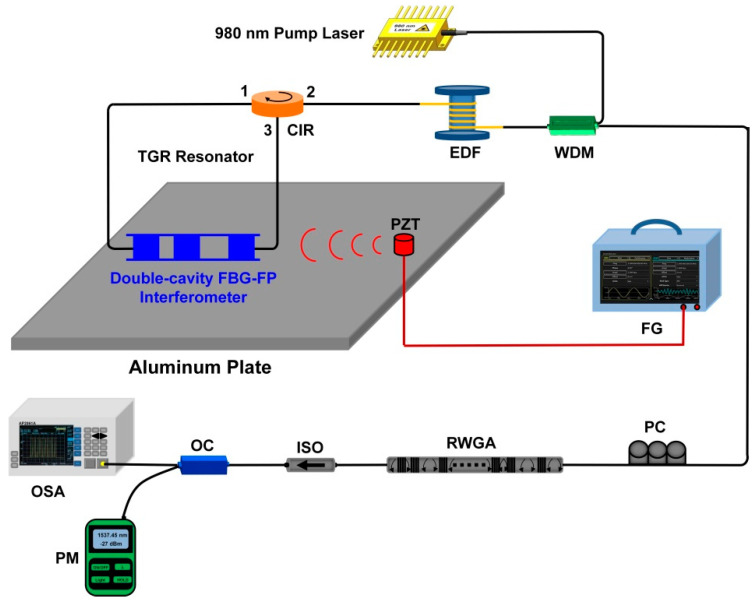
The experimental scheme of our proposed RFL. OC: optic coupler. OSA: optical spectrum analyzer. PM: power meter.

The experimental scheme of the narrow linewidth half-open-cavity RFL with a TGR resonator for dynamic strain detection is shown in Figure 1. A 980 nm source pumped the 4 m erbium-doped fiber through a 980/1550 nm wavelength division multiplexer (WDM), which provided sufficient linear gain. The RWGA provided high random feedback efficiency, where 30 weak FBGs were etched into an SMF through the phase mask method and ultraviolet exposure, which ensured the realization of multiple stabilized narrow-linewidth RFLs with a highly consistent performance. The reflectivity, central wavelength, and length of each weak grating were controlled to about 4%, 1537.5 nm, and 3 mm. The intervals between neighboring weak gratings were randomly selected in the range of 3–8 cm, which were the same as the following simulated parameter settings. The effective length of the RWGA feedback was about 167 cm. The proposed TGR resonator consisted of the specially designed double-cavity FBG-FP interferometer, a section of SMF, and a circulator (CIR). The CIR can maintain light unidirectional transmission and further suppress the spatial hole-burning effect. The designed double-cavity FBG-FP interferometer, which has one ultra-narrow peak, was fabricated by the same writing setup as RWGA. For effectively selecting lasing spectra, the wavelength of a single transmission window of our proposed TGR resonator should be within the 3 dB bandwidth of the reflection spectrum of RWGA. This high-reflectivity TGR resonator and RWGA feedback form a half-open-cavity structure, which can greatly improve the lasing efficiency of the RFL combined with the linear high gain of EDF. Additionally, the designed double-cavity FBG-FP interferometer was introduced to lock the laser and acted as a strain-sensing element. As shown in Figure 1, the double-cavity FBG-FP interferometer was bonded to the surface of aluminum plate. The size of the aluminum plate was 500 mm × 500 mm × 4 mm. A piezoactuator (PZT) actuator was bonded 5 cm away from the double-cavity FBG-FP interferometer. The sinusoidal signal with a 10 V voltage was applied from the function generator (FG) to the PZT actuator. The polarization controller (PC) adjusted the intensity and polarization states of light and minimized interference-related background noise. The isolator (ISO) mainly reduced the external Fresnel reflection and ensured that effective random feedback was only introduced by RWGA. The output spectrum and its peak-power were monitored by an optical spectrum analyzer (APEX, AP2061A, respectively). The total output power was measured by a power meter (OLP-6, Acterna).

### 2.2. Operation Principle of TGR Resonator

**Figure 2:**
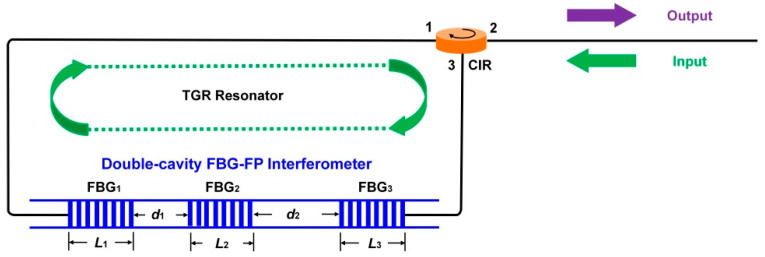
Schematic diagram of the proposed TGR resonator.

Figure 2 shows the schematic diagram of our proposed TGR resonator. The TGR resonator is composed of a double-cavity FBG-FP interferometer, a section of single-mode fiber and a circulator. The double-cavity FBG-FP interferometer can be considered as a grating connecting a SMF, as well as then connecting another grating. The double-cavity FBG-FP interferometer is embedded inside the TGR resonator. We can use a transfer matrix model to analyze its principle and transmission characteristics [31,32].

*F_i_*, *M_i_*, and *G* are the transfer matrix of FBG, SMF, and double-cavity FBG-FP interferometer, respectively, and can be expressed by Equations (1)–(3):

(1) $$F_{i}=\left[\begin{array}{cc} \cosh(SL_{i})-j\frac{\delta \sinh(SL_{i})}{S} & -j\frac{\kappa \sinh(SL_{i})}{S}\\ j\frac{\kappa \sinh(SL_{i})}{S} & \cosh(SL)+j\frac{\delta \sinh(SL_{i})}{S} \end{array}\right]$$

In Equation (1), *L_i_* is the length of each FBG, *δ* = *β*_1_ − *β*_2_ − 2π/*Λ*, *β*_1_ and *β*_2_ are propagation constant of forward and backward propagating field modes, respectively, *Λ* is the period of each FBG. *κ* is the coupling coefficient of the modes in each FBG, and *S*^2^ = *κ*^2^ − *δ*^2^.

(2) $$M_{i}=\left[\begin{array}{cc} \exp(jKd_{i}) & 0\\ 0 & \exp(-jKd_{i}) \end{array}\right]$$

In Equation (2), *K* = 2π*n_eff_*/*λ*, *n_eff_* is the refractive index of each FBG. *d_i_* is the length of SMF between two neighbor FBGs.

(3) $$G=F_{1}M_{1}F_{2}M_{2}F_{3}=\left(\begin{array}{cc} G_{11} & G_{12}\\ G_{21} & G_{22} \end{array}\right)$$

Based on Equations (1)–(3), the transmission coefficient of the double-cavity FBG-FP interferometer, *T_FBG-FP_*, can be expressed by:

(4) $$T_{FBG-FP}={\left|\frac{1}{G_{11}}\right|}^{2}$$

When simultaneously satisfying Equations (5) and (6), the single transmission peak of the double-cavity FBG-FP interferometer can appear at the central wavelength *λ_B_* [33]. The filtering performance can be improved by increasing the total cavity length of the double-cavity FBG-FP interferometer [34]. *R*_1_, *R*_2_, and *R*_3_ represent the reflection coefficient of the three FBGs, respectively. Making *R*_1_ = *R*_3_, the general condition for unity transmission can be expressed by:

(5) $$R_{2}=\frac{4R_{1}}{{\left(1+R_{1}\right)}^{2}}=\frac{4R_{3}}{{\left(1+R_{3}\right)}^{2}}$$

The length of cavities of the double-cavity FBG-FP interferometer *d*_1_ and *d*_2_ need to meet Equation (6):

(6) $$d_{1}=\frac{\left(2p+1\right)\lambda_{B}}{4n_{eff}},d_{2}=\frac{\left(2q+1\right)\lambda_{B}}{4n_{eff}}$$

*p* and *q* are arbitrary positive integers. *R*_1_, *R*_2_, and *R*_3_ are mainly determined by length *L*_1_, *L*_2_, and *L*_3_ and index modulation depth ∆*n*_1_, ∆*n*_2_, and ∆*n*_3_ of each FBG [35]. The effective refractive index of SMF is *n_eff_* = 1.46. The same central wavelength of each FBG is *λ_B_* = 1537.43 nm.

The fiber loop mirror (formed by a CIR and SMF) can maintain the unidirectional transmission of light and provide total reflection. Its transmission coefficient, *T_CIR_*, is about 100% in our proposed configuration. Thus, based on Equations (1)–(7), the transmission coefficient of our proposed TGR resonator, *T_TGR_*, can be expressed by:

(7) $$T_{TGR}=T_{FBG-FP}\cdot T_{CIR}$$

**Figure 3:**
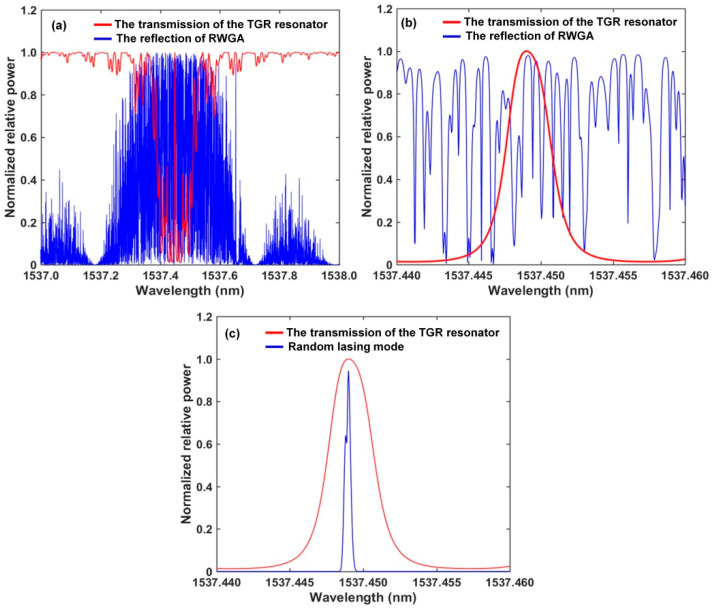
(**a**) The calculated transmission spectrum of TGR resonator and reflection spectra of RWGA; (**b**) enlargement of Figure 3a; (**c**) narrow lasing with multiple time gain.

Figure 3 shows the simulation results. The blue curve in Figure 3a is the calculated reflection spectrum of RWGA and consists of the previous results [32,35]. A lot of randomly scattered lights overlap for each other and this is mainly caused by many weak FBG pairs inside RWGA. To a certain extent, RWGA can be regarded as the envelope of narrow-band peaks superimposed by those weak FBG pairs as well as the result of a lot of random local modes. The calculated transmission spectrum (red curve) of the TGR resonator is shown in Figure 3a based on Equations (1)–(7). *L*_1_ = *L*_2_ = *L*_3_ = 10 mm, ∆*n*_1_ = ∆*n*_3_ = 4 × 10^−5^, ∆*n*_2_ = 8 × 10^−4^, and *R*_1_ = *R*_3_ = 45.38%, *R*_2_ = 85.88%. The sub-cavity length *d*_1_ = 18 mm, *d*_2_ = 54 mm. The TGR resonator has a narrow single transmission peak and flat loss outside the transmission peak. For effective filtering, the central wavelength of the transmission window should be within the 3 dB bandwidth of the reflection spectrum of RWGA. As shown in Figure 3b, the result shows that the TGR resonator can be used as the optical filter; a few random modes near the transmission window of the TGR resonator will obtain effective gain, overcome loss, and are expected to realize a narrow single-wavelength laser. The above random modes are amplified many times, and just the random mode near the central wavelength of the TGR resonator can obtain effective gain and become the dominant lasing mode. Figure 3c shows the simulated narrow single-wavelength lasing spectrum of the proposed RFL. The output wavelength of the simulated spectrum corresponds to the central wavelength of the transmission peak of the TGR resonator. The lasing spectral envelope of the RFL tends to be narrowed with the increased round-trip number of gain, which could be justified qualitatively through a process of coherence enhancement of the intra-cavity light through random feedback.

### 2.3. Linewidth Compression Principle of the Half-Open-Cavity of RFL

In the random resonant structure of RFL, the photon lifetime, *τ*, can be expressed by [36]:

(8) $$\tau =\frac{2n_{eff}}{c\alpha_{l}}l=\frac{2n_{eff}}{c\alpha_{l}}mL_{RFL}$$

*n_eff_* represents the effective refractive index of SMF. *c* is the speed of light in vacuum. *α_l_* represents the loss experienced by the photon for one round trip. *L_RFL_* represents the total length of the random resonator of the RFL. *l* represents the effective optical path of the photon round trip. *m* represents the number of round trips for photon.

The *Q* value of a random resonator can be expressed by [36]:

(9) $$Q=2\pi {\left[1-\exp\left(-\frac{T_{B}}{\tau }\right)\right]}^{-1}\approx \frac{2\pi \tau }{T_{B}}=2\pi \nu_{B}\tau$$

*T*_B_ represents the period of the photon round trip. Based on Equations (8) and (9), the 3 dB linewidth of the RFL can be expressed as:

(10) $$FWHM=\frac{\nu_{B}}{Q}=\frac{1}{2\pi \tau }=\frac{c\alpha_{l}}{4\pi n_{eff}L_{RFL}}\cdot \frac{1}{m}$$

When comparing the ring-cavity RFL with the same length of the RWGA feedback [11], the proposed RFL based on a half-open-cavity structure and a specially designed TGR interferometer exhibits the advantage of providing double optical gain in the EDF. The higher the gain efficiency of EDF, the higher the number of round trips for the photon. According to Equation (10), the 3 dB linewidth of the proposed RFL can become narrower with the increase of the number of round trips for the photon.

### 2.4. Strain-Sensing Principle of the Proposed RFL Sensor

The relative deviation of the double-cavity FBG-FP interferometer wavelength (frequency) is related to the applied dynamic strain, *ε*, by the following expression [37]:

(11) $$\frac{\Delta \lambda_{B}}{\lambda_{B}}=\frac{\Delta \nu_{B}}{\nu_{B}}=K\Delta \varepsilon$$

where *ν_B_* is the double-cavity FBG-FP interferometer resonance frequency (wavelength), Δ*ν_B_* is frequency (wavelength) deviation induced by the applied dynamic strain, *K* ≈ 0.78 is a constant related to the Poisson’s ratio and Pockels coefficients of the photo-elastic tensor of the fiber glass.

The power spectral density *S_ε_*(*f*) of the applied strain in the double-cavity FBG-FP interferometer (embedded inside the RFL sensor) is given by [37]:

(12) $$S_{\varepsilon }(f)=\frac{S_{\Delta \nu_{B}}(f)}{\nu_{B}K}=S_{\Delta \nu_{B}}(f)\cdot \frac{\Delta \varepsilon }{\Delta \nu_{B}}\left[\varepsilon /Hz\right]$$

where *S*_Δ__ν__B_(*f*) is the power spectral density of the frequency fluctuations between the RFL and the double-cavity FBG-FP interferometer resonance frequencies. Considering the conversion factor of Δ*ε*/Δ*ν_B_* = 6.6 × 10^−15^ *ε*/Hz [38].

In the high frequency domain, the frequency noise *S*_Δ_*_ν__B_*(*f*) can be estimated by the 3 dB linewidth (*FWHM*) [39]:

(13) $$S_{\Delta \nu_{B}}(f)=\sqrt{\frac{FWHM}{\pi }}$$

Based on Equations (12) and (13), the power spectral density *S_ε_*(*f*) of the applied strain can be estimated by the 3 dB linewidth (*FWHM*):

(14) $$S_{\varepsilon }(f)=6.6\times 10^{-15}\sqrt{\frac{FWHM}{\pi }}$$

The narrow linewidth is beneficial to suppress frequency noise levels and can further improve the resolution of the RFL dynamic strain sensor [3]. RFLs have a simple cavity configuration, low threshold and good temporal coherence, and thus, are promising alternatives for narrow linewidth and low frequency noise RFL sensors [40].

## 3. Experimental Results and Discussion

### 3.1. Output Charateristics of RFL

**Figure 4:**
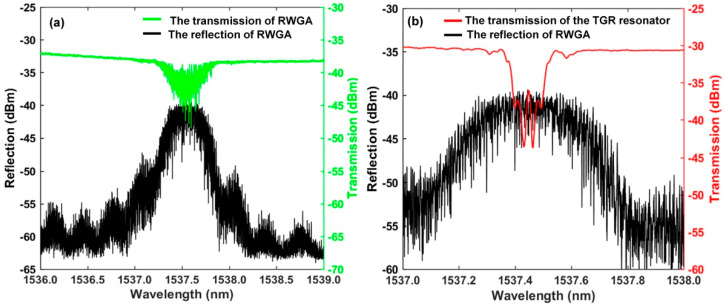
(**a**) Reflection and transmission spectrum of RWGA; (**b**) reflection and transmission spectrum of the TGR resonator and RWGA.

We used an OSA with a resolution of 1.12 pm to measure the spectra of RWGA. Figure 4a shows that the RWGA has a 3 dB bandwidth of 0.393 nm and a central wavelength of 1537.45 nm. The transmittance, *T_RWGA_*, of the RWGA is about 13%. The random fiber feedback mechanism is mainly provided by the photon localization effect inside RWGA. Random lasing is expected as long as the photon localization length is shorter than effective length of RWGA. The effective length of the RWGA, *L_RWGA_*, is about 167 cm. The localization length, *ξ*, can be estimated from *ξ* ≈ *L_RWGA_*/2ln(1/*T_RWGA_*) [41]. The calculated photon localization length is about 40.6 cm and is shorter than the effective length of RWGA. The random lasing and photon localization effect will exist in the proposed RFL. As shown in Figure 4b, the measured spectra are similar to the simulated output spectra in Figure 3a, and the measured central wavelength of the TGR resonator is 1537.426 nm. For effectively selecting lasing spectra, the wavelength of the single transmission peak of the proposed TGR resonator should be within the 3 dB bandwidth of reflection spectrum of RWGA. A lot of disordered narrow-band random peaks can be found in the reflection spectrum of RWGA, which are mainly caused by multiple reflection and interference formed in many weak grating pairs. Besides, a lot of randomly scattered lights overlap with each other as well as result in a lot of random local modes. These random local modes are amplified many times, and a few random modes near the central wavelength of the TGR resonator overcome the loss of the random cavity and achieve narrow linewidth lasing. Furthermore, increasing the number and randomness of the weak grating pair inside RWGA is beneficial to compress the linewidth of RFL.

**Figure 5:**
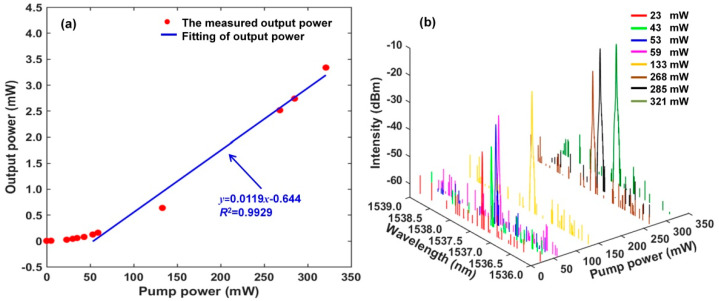
(**a**) Output power versus pump power; (**b**) output spectrum changes with pump power.

Figure 5a shows the trend of total output power of our proposed narrow linewidth RFL as a function of pump power. Due to the low insertion loss of TGR resonator, double optical gain of EDF and high random feedback efficiency of RWGA, a low lasing threshold of about 23 mW is achieved in our proposed RFL. The total output power of the RFL increases with the pump power. The linearity of about 99.29% and the slope efficiency of about 1.19% are calculated respectively. When pump power increases to 321 mW, the maximum output power of 3.34 mW is obtained. The lasing spectra of our proposed RFL based on the TGR resonator as a function of the pump power are shown in Figure 5b. It can be seen that the lasing spectrum does not appear bellow the lasing threshold of 23 mW, because the gain of EDF is insufficient for the random oscillating modes to overcome losses in the random resonant cavity. Meanwhile, when the pump power exceeds lasing threshold, a narrow linewidth single-wavelength lasing spectrum at 1537.426 nm is realized. The central wavelength of lasing spectrum remains at the 1537.426 nm with the pump power increasing, which indicates that our proposed narrow linewidth RFL based on TGR resonator can easily realize stable single−wavelength operation as the pump power exceeds lasing threshold.

**Figure 6:**
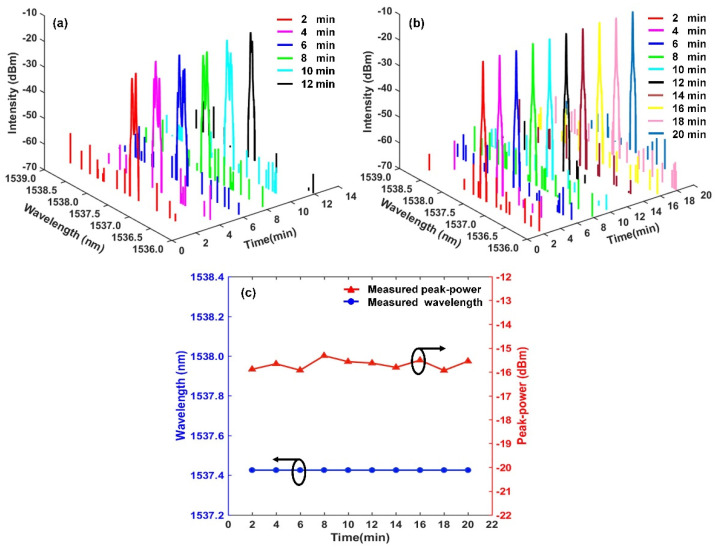
Time-stability test of lasing spectra of proposed RFL. (**a**) Without TGR resonator; (**b**) with TGR resonator; (**c**) stability of lasing wavelength and output power at pump power of 200 mW.

When the pump power is fixed at 200 mW, the time−varying of output spectra of our proposed RFL without and with the TGR resonator are shown in Figure 6a,b. The different colors spectral lines are shown in Figure 6a, with time, there is no stable single−wavelength lasing without a TGR resonator. Under the flat linear gain of EDF, the gain competition of random resonant−cavity modes is serious due to their approximate quality factor. Thus, a few of the random resonant−cavity modes are simultaneously amplified and oscillated, which lead to random splitting lasing spectra. Multitudinous random sub−cavity modes are induced into the RFL as the pump power increases. Those random resonant modes with low quality factors are also activated and emitted, which easily results in multi−spike lasing of the output spectrum and spectral broadening. Besides, nonlinear effects inside RWGA, broadband linear gain of EDF, and energy fluctuations in the random resonant cavity also contribute to the unstable output spectrum with time. As shown in Figure 6b, when the designed TGR resonator is embedded into the RFL, within 20 min (min), a long−term stable single−wavelength lasing spectrum without obvious fluctuations is achieved. The OSNR of the stable lasing spectrum at the central wavelength of 1537.426 nm is up to about 43 dB. The proposed TGR resonator just accommodates dominated random sub−cavity modes inside RWGA and suppresses undesired residual modes hopping. The dominant position of the random lasing mode is enhanced, resulting in a stable single−wavelength output. Figure 6c shows the variation of the central wavelength and output peak−power with time. The maximum fluctuation of the lasing wavelength is less than 1 pm. The maximum fluctuation of the output peak−power is about 0.62 dB. The output wavelength and peak−power exhibit good long-term stability. The TGR resonator inside the proposed RFL can effectively reduce the amount of resonant modes and adjust the transmission loss of the random resonant cavity. The lasing wavelength is locked in the narrow transmission window of the TGR resonator, ensuring a relatively stable output. Besides, the higher stability of the pump source can contribute to the time−domain stability of the output spectrum. The experimental results clearly indicate that the proposed TGR resonator can bring the RFL from an unstable multi−wavelength to a stable single-wavelength operation.

### 3.2. Frequency Characteristics of RFL

**Figure 7:**
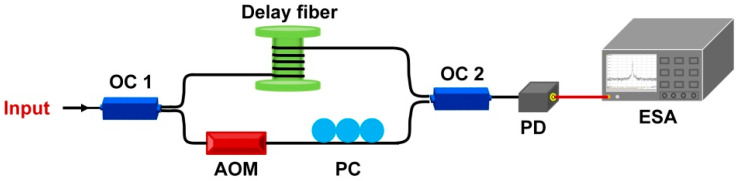
DSH method for the linewidth measurement of RFL.

The linewidth measurement of the RFL based on delayed self−heterodyne (DSH) technique is shown in Figure 7. The OC 1 has a ratio of 95/5 and divides the output light of the RFL into two parts, with the larger part going into the delay fiber and the smaller part entering an acousto−optic modulator (AOM) and a PC, which downshifts the optical frequency of the laser light by 200 MHz. A photo detector (PD) is connected to the recombination of two output light beams to detect the beat spectrum that centered at 200 MHz with an electrical spectrum analyzer (ESA). The length of the delay fiber is controlled with 75 km to ensure that two output light beams are uncorrelated with each other. Then, an accurate Lorentzian line shape measurement of RFL is recombined and obtained.

**Figure 8:**
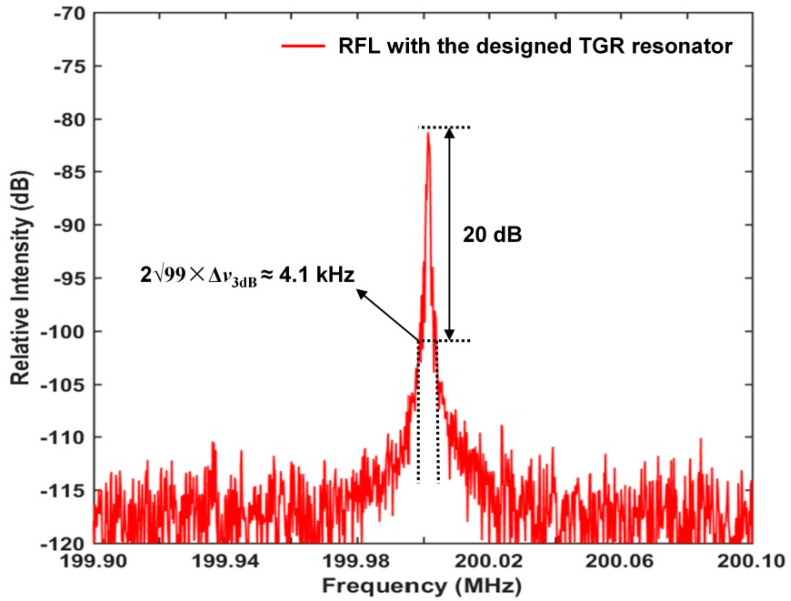
Power spectrum of the beat signal normalized to 200 MHz.

Figure 8 shows that the measured 20 dB beat signal linewidth of the RFL is about 4.1 kHz, corresponding to the 3 dB Lorentzian linewidth of about 205 Hz. Just a single lasing peak is observed, which indicates the effective suppression of undesired residual random lasing modes by the proposed TGR resonator. Usually, a much narrower linewidth of the laser contributes to achieve lower frequency noise as well as a higher dynamic-strain resolution in the high frequency range. According to Equation (14), the dynamic strain resolution of our proposed RFL strain sensor is estimated to be 53 fε∕√Hz through the measured linewidth. It is worth noting that the linewidth is not a major determinant of frequency noise of the RFL.

**Figure 9:**
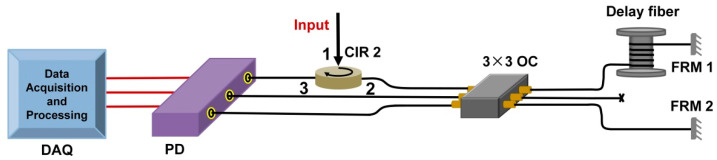
High speed interrogation based on 3 × 3 coupler for frequency noise measurement. DAQ: data acquisition equipment.

The frequency noise of the narrow half−open−cavity RFL is measured by using a 3 × 3 unbalanced fiber Michelson interferometer and a digital phase demodulation scheme [42], as shown in Figure 9. The output light enters the unbalanced fiber Michelson interferometer (MI) via a 3 × 3 OC and a CIR, where a section of conventional forty−five−meter−long SMF is inserted into one arm as a delay fiber. The polarization fluctuation inside the MI is eliminated by two Faraday rotating mirrors (FRMs). Then, three output lights of the 3 × 3 OC go into the multi−channel photo detector (KG−PR−1M−A−FC, manufactured by Conquer, China) and are then converted into electrical signals. An analog−to−digital card with a response bandwidth of 1 MHz is used to acquire electrical signals. For extracting the phase information of the fiber MI and precisely interrogating wavelength under high−speed sampling, the interrogation algorithm is written into a Xilinx field programmable gate array (NI FlexRIO) development board and implemented effectively. The highly stable 980 nm pump laser and our proposed RFL are put in two sealed stainless steel boxes with vibration and sound isolation cotton to suppress environmental noise and keep a relatively constant temperature.

**Figure 10:**
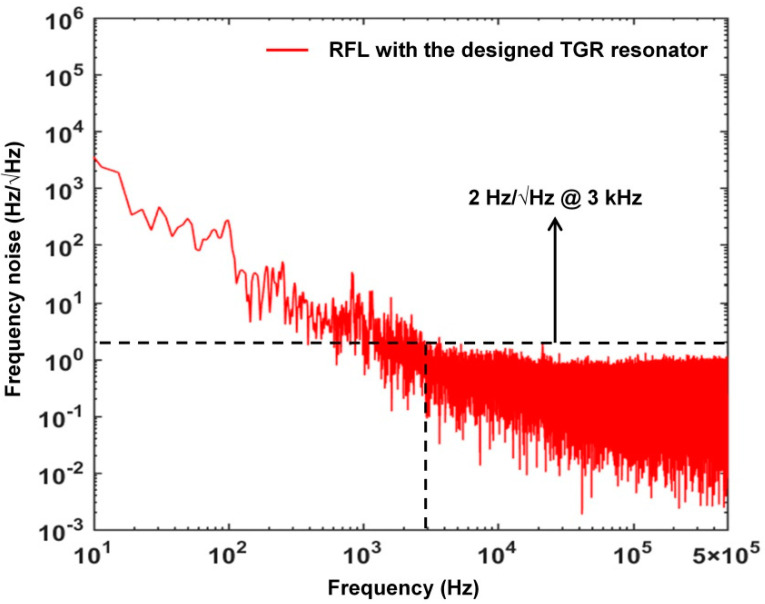
Measured frequency noise of the proposed RFL with a TGR resonator.

Usually, a narrow linewidth of a laser means that the low frequency noise can be realized, which contributes to improve the strain resolution in a high frequency range. It is worth noting that the linewidth is not a major determinant of frequency noise of the RFL. The thermally induced frequency noise is from random thermal fluctuations in the fiber. In Figure 10, the frequency noise of our proposed narrow linewidth half-open-cavity RFL with the TGR resonator is reduced to 2 Hz/Hz above 3 kHz. Such a low noise level benefits from the effective suppression of competition-induced frequency jitter and residual undesired random lasing modes by the TGR resonator. The random distributed feedback provides a Lorentzian envelope over the original laser frequency noise, which has a significant impact on the reduction of thermal−induced frequency noise. Furthermore, the half−open−cavity structure can provide double higher optical gain of EDF, which is beneficial to increase the photon lifetime, produce a narrower linewidth, and further suppress frequency noise. Additionally, it is seen that the frequency noise spectrum of our proposed RFL shows a relatively flat and low noise level in the high frequency range. However, a hopping peak with low amplitude occurs at 2.5 kHz, which is mainly caused by the intrinsic relaxation oscillation of EDF. Usually, the dynamic strain measuring resolution of RFL sensors can be obtained when the response of the strain signal is equal to the noise floor of sensing system.

### 3.3. Strain Detection Characteristics of RFL

**Figure 11:**
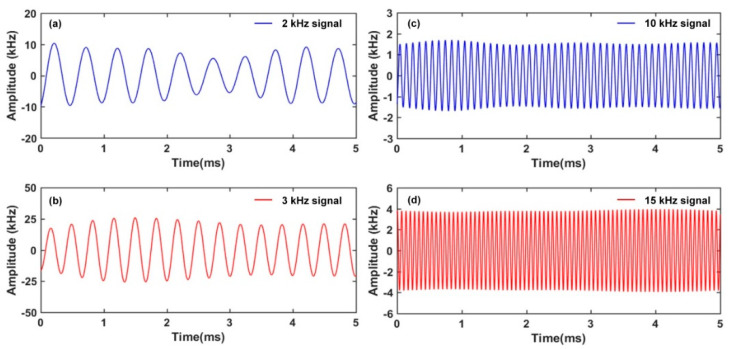
The response of RLF sensor to continuous dynamic strain signal.

The response of the RFL sensor to dynamic strain signal is further investigated. The designed double−cavity FBG-FP interferometer is inserted into the proposed TGR resonator for locking the laser and acting as a strain sensing element. As shown in Figure 1, the double−cavity FBG-FP interferometer is bonded to the surface of aluminum plate. The size of aluminum plate is 500 mm × 500 mm × 5 mm. The PZT actuator is bonded with 6 cm away from the double−cavity FBG-FP interferometer. The sinusoidal signal with a 10 V voltage is applied from the function generator (FG) to the PZT actuator. A dynamic strain signal with up to kilohertz frequency can be generated and then propagate along the aluminum plate. Such dynamic strain signal disturbs the index modulation planes of the double−cavity FBG-FP interferometer, which introduces a frequency shift of reflection spectrum. As a consequence, the round−trip random cavity loss caused by the dynamic strain signal will impose an intensity modulation of the random fiber laser. According to Figure 1, the FG drives PZT to generate a sinusoidal signal with a 10 V voltage to simulate the strain source. The low−frequency interference inside the original signal is eliminated through a filter with 1 kHz threshold. As shown in Figure 11a–d, laser modulation that is induced by different strain signals of 2 kHz, 3 kHz, 10 kHz, and 15 kHz is recorded. The periodic sinusoidal strain signals present a good waveform.

**Figure 12:**
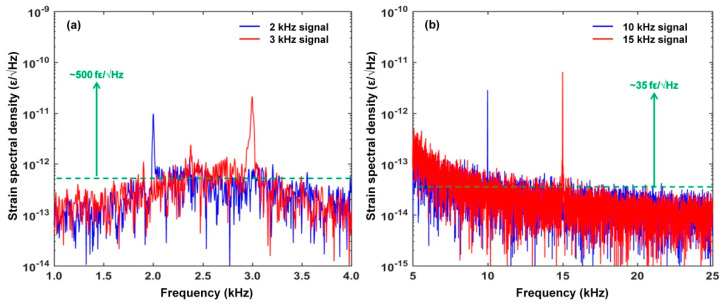
The power spectral density of measured dynamic strain signal.

Figure 12 shows the strain power spectral density of the response of our proposed RFL sensor. In accordance with 1.21 pm/με sensitivity [43], as shown in Figure 12a, the dominating signal peaks are found at the frequency of 2 kHz and 3 kHz, respectively, and the resolution of the dynamic strain is about 500 fε/√Hz. As shown in Figure 12b, the dominating signal peaks are found at the frequency of 10 kHz and 15 kHz, respectively, and the resolution of the dynamic strain is about 35 fε/√Hz. The measured resolution of 35 fε/√Hz corresponds to the estimation of the dynamic strain resolution (53 fε/Hz) calculated by the 3 dB linewidth. This clearly indicates that the proposed RFL strain sensor provided a good response with the identical frequency to the generated dynamic−strain signal. By adjusting the PZT driving frequency of the sinusoidal signal from the function generator, kHz continuous dynamic strain signal can be activated and then presented. This experimental result suggests that our proposed RFL strain sensor based on a double−cavity FBG-FP interferometer is capable of dynamic strain detection. We note that the relatively lower dynamic strain resolution at frequencies of 2 kHz and 3 kHz mainly resulted from the higher laser intensity fluctuation background at intrinsic frequencies close to about 2.5 kHz. Furthermore, the hopping peak with a low amplitude was caused by the intrinsic relaxation oscillation of the EDF−based RFL. Thanks to the narrower linewidth and lower frequency noise, our proposed RFL strain sensor exhibits about one−order of magnitude enhancement of the dynamic−strain resolution compared to the π−FBG−based random fiber laser strain sensor with a half−open−cavity structure [30].

In Table 1, a performance comparison of a narrow random fiber laser dynamic−strain sensor is presented. Our proposed RFL sensor has apparent advantages in dynamic strain resolution. The sensing system maintains a relatively minimum resolution of about 35 fε/√Hz. The specially designed double−cavity FBG-FP interferometer, which acts as a sensing probe, can contribute to improve the dynamic strain resolution.

## 4. Conclusions

A stabilized narrow linewidth random fiber laser for strain detection, based on a TGR resonator and half−open−cavity structure, was proposed and investigated experimentally. The half-open-cavity structure was proven to provide double higher optical gain of EDF, which is beneficial to increase the photon lifetime and further narrower the linewidth. Furthermore, the proposed TGR resonator is composed of a double−cavity FBG-FP interferometer, a section of SMF, and a circulator. The specially designed double−cavity FBG-FP interferometer embedded in the TGR resonator acted as the strain sensing element. Meanwhile, the TGR resonator could effectively suppress the competition−induced undesired multi−spike lasing, which contributed to establishing a stabilized single−wavelength lasing spectrum, further suppress the frequency noise of RFL, and improve the resolution of dynamic strain. A stable ultra−narrow linewidth of about 205 Hz was obtained. The frequency noise was reduced to about 2 Hz/√Hz. A high dynamic strain measuring resolution of 35 fε/√Hz was achieved ultimately. This provides a novel choice for weak dynamic strain signal detection in structural health monitoring and so on.

## Figures and Tables

**Table 1:** Performance comparison of RFL dynamic strain sensor.

Cavity Type	Sensing Structure	Resolution	References
Ring	Random grating array	~720 fε/√Hz	[29]
Half−open−cavity	π−FBG	~280 fε/√Hz	[31]
Ring	π−FBG	~140 fε/√Hz	[28]
Half−open−cavity	double−cavity FBG-FP ^1^	~35 fε/√Hz	This work

^1^ The double−cavity FBG-FP interferometer is embedded in the proposed TGR resonator.

## Data Availability

The data are not publicly available due to the Confidentiality and Non-disclosure Agreement with the funders.
